# Clinical vitamin A deficiency among preschool aged children in southwest Ethiopia

**DOI:** 10.3389/fnut.2024.1267979

**Published:** 2024-02-21

**Authors:** Abdilwahid Nuredin, Tamirat Melis, Abdu Oumer Abdu

**Affiliations:** ^1^College of Medicine and Health Sciences, Wolkite University, Wolkite, Ethiopia; ^2^School of Public Health, College of Medicine and Health Sciences, Haramaya University, Harar, Ethiopia

**Keywords:** bitot’s spots, clinical vitamin A deficiency, conjunctival xerosis, determinants, night blindness, preschool age children

## Abstract

**Background:**

The clinical manifestations of vitamin A deficiency (VAD) involve night blindness, bitot’s spots, corneal xerosis, and corneal scars. It is the most important cause of preventable childhood blindness among children and causes morbidity and mortality. Even though Ethiopia implemented high-potency vitamin A supplements, the occurrence of VAD remains significant. This study was to identify determinants of clinical VAD among preschool-aged children (PSC) in southwest Ethiopia.

**Method:**

A community-based survey was conducted among 411 randomly selected PSCs. A pretested and structured questionnaire coupled with clinical observation for signs of vitamin A deficiency by a trained ophthalmologist was used to collect the data. An anthropometric measurement of height was taken and analyzed using WHO Anthro to calculate Z-scores for each index. The public health significance of VAD was declared after comparison with international references. A bi-variable and multi-variable logistic analysis was done. We reported the adjusted odds ratio (AOR), 95% confidence interval, and *p*-value.

**Result:**

A total of 411 children were screened for clinical VAD, and the overall prevalence was 2.2% (95% CI: 1.5–2.5). Of which, night blindness affects 1.2%, bitot’s spots affects 0.7%, and corneal xerosis affects 0.2%, indicating a major public health problem compared to the international reference. The odds of clinical VAD were 81% lower among children who received vitamin A supplementation (VAS; AOR = 0.19, 95% CI: 0.04–0.92). On the other hand, PSC of mothers who had attended ANC visits were 89% less likely to develop clinical VAD (AOR = 0.11, 95% CI: 0.02–0.53). In addition, the study revealed that the odds of developing clinical VAD are 82% lower among PSC aged 36 to 47 months (AOR = 0.18; 95% CI: 0.03–0.97).

**Conclusion:**

The prevalence of clinical VAD among PSC is a public health problem and is associated with ANC visits, VAS status, and the age of the child, which could be used to target interventions to further reduce existing VAD. Further studies using reliable dietary intake and biomarker data could further depict the burden of subclinical VAD.

## Introduction

Vitamin A plays a fundamental role in various body functions, including vision, growth, immunity, reproduction, the integrity of epithelial cells, and survival (1). Hence, it is an essential nutrient that cannot be synthesized by the body and should be taken from the diet (2). The average child should consume 500–700 mcg of vitamin A daily to prevent the risk of vitamin A deficiency (VAD) (3, 4). Hence, the World Health Organization (WHO) recommends biannual supplementation of children with 30–60 mg of retinol equivalent to reduce child morbidity and mortality (5, 6).

VAD is a critical concern for children under five, especially in developing countries. To address this issue, a comprehensive dietary approach is necessary. Animal source foods like milk and eggs, which provide retinol directly, are crucial in alleviating VAD in regions with high VAD prevalence (7, 8). Additionally, incorporating orange-fleshed sweet potato (OFSP) into diets can serve as a complementary strategy, as they are rich in beta-carotene, a precursor to retinol (7, 9). However, limited access to both animal source foods and OFSP in low-income areas heightens the risk of VAD, highlighting the urgent need to improve availability and affordability of these vital food sources (8).

Vitamin A Deficiency (VAD) is characterized by various eye manifestations, collectively known as xerophthalmia (10, 11). These manifestations include night blindness, bitot’s spots, conjunctival xerosis, corneal dryness, and in severe cases, the destruction of the eyeball leading to blindness. The progression of these symptoms follows a gradual pattern in the natural history of VAD. It typically starts with reduced vision in low-light conditions (night blindness), followed by dryness of the conjunctival mucosa and the appearance of foamy, whitish-gray patches on the conjunctiva (bitot’s spots). As the deficiency worsens, the cornea becomes affected, resulting in corneal ulcers, scarring, and eventual loss of the eyeball and vision (xerophthalmia) (4).

VAD is characterized by xeropthalmia, eye manifestations of VAD include night blindness, bitot’s spots, conjunctival xerosis, corneal dryness, and destruction of the eyeball leading to blindness (10, 11). These are gradual symptoms of VAD in the natural history where the child can have reduced vision at dark (night blindness), followed by dryness of the conjunctival mucosa and accumulation of keratin in the conjunctiva (bitot’s spot), involvement of the cornea (corneal ulcer and scaring) and gradual loss of eye ball and vision at the end (xerophthalmia). Some of these manifestations could be averted through vitamin A supplementation (VAS) (3, 12).

Still, VAD is a major nutritional concern in lower-income countries (11). Both subclinical and clinical VAD are the most important causes of preventable childhood blindness, especially among children (1, 3, 4). Low intake of vitamin A during critical periods, coupled with common childhood illnesses, increases the risk of developing clinical VAD (4). The WHO report showed that VAD affects up to one-third of Preschool-age children (PSC) and contributes to 250, 000–500, 000 cases of blindness (12). It is a severe public health problem in more than 120 countries in the world (3).

Although the prevalence of VAD has declined from 39% in 1991 to 29% globally, the highest prevalence still exists in sub-Saharan Africa. Moreover, VAD accounts for 1.5% of deaths, and 95% of these deaths are recorded in SS, including Ethiopia (48%) (13). In Ethiopia, VAD leads to 80,000 deaths per year and affects 61% of PSC (14). The risk of VAD reaches 60.3%, and it is closely correlated with food consumption patterns (15). More importantly, maternal vitamin A status and the amount of retinol in breast milk are extremely low, predisposing children to increased risk (16). In addition, 1.7% and 0.8% of children had bitot’s spots and night blindness, respectively, indicating significant public health concerns (17). Improving vitamin A status could significantly reduce the risk of mortality from measles by 50%, from diarrhea by 40%, and overall mortality by 25–35% (12).

The practice of VAS can avert an estimated 167,563–376,030 child deaths annually, but the effective VAS coverage could reduce VAD and showed a great variation by wealth and other characteristics (18). Thus, the risks of VAD showed wide variability, mainly due to access to vitamin A-rich foods and VAS coverage (10, 16–18). Hence, the occurrence of VAD could vary from time-to-time between geographic locations and be affected by the season of the study. It could be helpful to study during a very lean season to capture the worst-case scenario for the occurrence of VAD in a population.

Several studies have identified important predictors of Vitamin A Deficiency (VAD) in Ethiopia, including maternal education, family size, droughts, respiratory or diarrheal illnesses, dietary diversity status, availability of latrines, income status, sex of the child, and stunted growth (12, 17, 19, 20). Vitamin A-rich food consumption in the country is seasonal, poor, and geographically clustered (21), and despite periodic high-potency vitamin A supplementation programs, clinical VAD remains a major public health problem (22–24). Access to and consumption of vitamin A-rich foods are low, particularly in southern regions (21), and affordability is decreasing (25). These challenges highlight the need for updated epidemiological evidence and the evaluation of existing interventions to address VAD effectively.

Although sub-clinical VAD could increase the risk of illness and VAS is in place in Ethiopia, the current study would be a valuable input in various ways. Firstly, this study aims to assess the efficacy of national policy and the vitamin A distribution program, partly evaluating the program’s impact on reducing vitamin A deficiency and its associated health issues. Secondly, identifying gaps and target populations despite the existence of a national program in place helps to identify high-risk groups and serves as a tool to pinpoint these gaps, facilitating targeted interventions in the study setting. Hence, the current study was to assess the magnitude of different forms of clinical VAD and its potential risk factors in the case of southwest Ethiopia.

## Materials and methods

### Study design and setting

A community-based survey was conducted during the lean season from March to April 2023 in Cheha district, located in southwest Ethiopia. Cheha is one of the districts found in the Gurage Zone, southwest Ethiopia. Agriculture is the backbone of their economy. Enset, corn, sorghum, chickpeas, teff, and niger seed are the most commonly eaten foods by the community in the district. The availability of vitamin A-rich fruits and vegetables is usually seasonal and limited (25). The district has 38 kebeles with a total of 26,405 households, six health centers, and 38 health posts. It has a total population of 137,574, of which 49.8% are males and 50.2% are females. The number of children under 5 years old is 21,479, out of which the number of children aged 3 to 5 years old is 10,042 (26).

### Population and eligibility

The study population includes all randomly selected eligible PSC aged 36 to 59 months and their mothers or caregivers who were living in randomly selected kebeles. Children who are aged 3 to 5 years (36 to 59 months) and their mother or caretaker who has a mental problem or is critically ill in the absence of a close caregiver were excluded. This study mainly targets children in this age group due to their increased susceptibility and suitability for community-based studies.

### Sample size determination

The required sample size was determined by using a single population proportion formula assuming a 95% confidence level, a prevalence of VAD of 2% in Farta district, Ethiopia, and a margin of error of 3% due to the rare nature of the outcome (27). The sample size was 92. Using the StatCalc package in EPiInfo software, the sample for the second objective was calculated. Thus, the sample size was determined by considering various factors that are significantly associated with the outcome variable at 80% power and 5% significance levels and considering the ANC visits of the mother (28) and head of household (29), determinant of clinical VAD. Accordingly, the minimum sample size estimated by ANC visits of mothers was 418 by adding the 10% non-response rate. Hence, a survey of about 418 children was required to assess the determinants of clinical VAD in the study area.

### Sampling procedure

A combination of stratified random sampling and systematic random sampling was used to elect the study participants. The 38 kebeles in Cheha district were stratified into seven stratums or clusters based on their geographical location. Then, a total of twelve kebeles were randomly selected from the seven strata. The required sample size was distributed to each of the twelve selected kebele based on proportional allocation, and the study units were selected using systematic random sampling techniques. The first household for the study was selected randomly, and then every third household was included in the study. Where there was no eligible child within the selected household, the adjacent household was visited, and when there was more than one eligible child in a given household, one child was randomly selected.

### Study variables

The dependent variable of this study is clinical VAD, which includes night blindness, conjunctival xerosis, corneal xerosis, corneal scars, corneal ulceration, corneal scar, and xeropthalmia. These were assessed by trained and experienced ophthalmology professionals using a history and physical examination. While the socio-demographic characteristics (age, sex, educational status, occupational status, religion, marital status, family size, and income) and health and nutritional factors (stunting, wasting, underweight, dietary diversity, vitamin A-rich vegetables, and child illness) and vaccination-related factors (vitamin A supplementation status and immunization status of the child) were considered independent variables of the current study.

### Data collection tools and techniques

Data was collected by three optometrist nurses and two Integrated Eye Care Workers (IECW). Two days of training were given for data collectors and supervisors on the objective of the study, the method of facilitating respondents, and the context of the questionnaire by the principal investigator. A structured and pretested questionnaire, along with clinical observation for signs and symptoms of clinical VAD traced by trained clinicians, was used to collect the data. The questionnaire was adapted from different relevant studies by the WHO and the Food and Agriculture Organization (FAO). First, it was developed in English and then translated into Amharic and the local language, “Guragegna,” and back translated to English to check its consistency. The weight and height of children were measured according to standard procedures. The weight of the child was measured using a calibrated electronic weight scale to the nearest 0.1 kg. The height of the child was measured with a standing wooden stadiometer to the nearest 0.1 cm. Height was measured without shoes in the Frankfurt position, where the line of sight was perpendicular to the measuring board. Based on the weight, height, and age of the child, the corresponding anthropometric indices Z-score (WAZ, HAZ, and BAZ) were calculated using the WHO Anthro software (30).

The validated 8-food group dietary diversity tool developed by FAO was used to assess the dietary diversity score of children. The dietary intake was collected over the past 24 h, excluding exceptional fasting and feasting days. These are very predictive of micronutrient adequacy levels (31) and the risk of micronutrient deficiencies, including VAD (31, 32).

### Operational definition

In this study, children who are 3 to 5 years (36 to 59 months) of age were considered as PSC. A child with clinical VAD has either a history of night blindness or has a bitot’s spots, conjunctival xerosis, corneal xerosis, corneal ulceration, or corneal scar upon examination. In addition, children with a dietary diversity score (DDS) of four or above in the last 24 h of the survey were classified as having a good or adequately diversified diet (33). The clinical signs and symptoms of Vitamin A Deficiency (VAD) in children in this study were assessed using the following operational definitions. Night blindness was defined as the child’s inability or difficulty seeing in low-light conditions, which was reported by caregivers. Conjunctival xerosis was identified through clinical examination and characterized by dryness and roughness of the conjunctiva, the clear membrane covering the white part of the eye. Bitot’s spots were described as foamy, whitish-gray patches on the conjunctiva resulting from keratin accumulation, also observed during clinical examination. Corneal xerosis, referring to the drying and clouding of the cornea, and corneal ulceration with scars were evaluated through physical examination conducted by experienced optometrists (3).

### Data processing and analysis

The data was edited, coded, and entered into Epi Data version 3.1 and exported to SPSS version 22 statistical software for analysis. After cleaning the data for inconsistencies and missing values in SPSS, descriptive statistical analysis such as mean, median, standard deviation, percent, and frequency was done. A bivariable logistic regression was performed for each independent variable and outcome variable. By considering the result of the bivariable analysis, variables were selected for the multivariable analysis to control for confounding. A variable whose bivariable test has a *p*-value of 0.20 was selected for the multivariable model. Variables that have higher co-linearity were excluded from the regression. Once the variables were identified, multivariable logistic regression analysis with a *p*-value of 0.05 and an AOR with a 95% CI was used to measure the degree of association between independent variables and the outcome variable (33). Multicollinearity among independent variables was evaluated using collinearity diagnostics (33). We have further potential interaction effects among the variables for better modeling (33).

### Data quality control measures

In order to assure the quality of the data, training was given to the data collectors on the objective of the study, the data collection process, and the relevance of the study prior to data collection. A pilot study was done before the actual data collection among 21 respondents (5% of the total sample size) on non-selected kebeles. Through the pilot study, the flow of the questions and language usage were modified for the actual data collection. During the data collection process, different WHO-standardized pictures for clinical signs of vitamin A deficiency were used as a golden standard for comparison. The data was checked for completeness, accuracy, and clarity on a daily basis. Throughout the course of the data collection, data collectors were supervised at each site with daily supportive feedback. The calibration of the weight scale and height scale was always checked before measuring every child’s weight and height, respectively. The anthropometric measurements were done using standardized procedures and methods (33).

### Ethical considerations

Ethical clearance was obtained from the Wolkite University Colleges of Medicine and Health Science, Research Ethics Committee, and a letter of permission was obtained from the Cheha district health office. The purpose of the study was explained to respondents, and verbal informed consent was obtained from the mothers and caregivers. The confidentiality of the information was maintained by omitting any personal identification from the questionnaires. Respondents were informed about the study and the variety of information needed from them. During the data collection process, those children showing signs of clinical VAD were given a therapeutic dose of vitamin A according to the guidelines. A chance was given to the respondent to ask anything about the study, and the right not to participate in the study was kept at any moment.

## Results

### Socio-demographic characteristics

A total of four hundred eleven (411) children and their mothers or caregivers participated in the study, making the response rate 98.3%. About 215 (52.3%) of the children were female. About 401 (97.6%) households had less than three children under 5 years old. In the majority, 400 (97.3%) of the respondents were married, 166 (40.4%) of the mothers and caregivers did not attend formal education, and 182 (44.3%) attended primary education. About 252 (61.3%) children were aged 36–47 months (Table 1).

**Table 1:** Socio-demographic characteristics of study participants in Cheha District, southern Ethiopia, 2023.

Characteristics of mother/caregiver	Category	*n* (%)
Mother/caregiver age	≤35	344 (83.7%)
	>35	67 (16.3%)
Mother/caregiver marital status	Married	400 (97,3)
	Divorced	6 (1.5%)
	Widowed	5 (1.2%)
Mother/caregiver occupation	Housewife	294 (71.5%)
	Merchant	69 (16.8%)
	Government employer	22 (5.4%)
	Daily labor	13 (3.2%)
	Private employer	12 (2.9%)
	Farmer	1 (0.2%)
Mother/caregiver educational status	Not attend formal education	166 (40.4%)
	Primary education	182 (44.3%)
	Secondary education	44 (10.7%)
	Collages and above	19 (4.6%)
Monthly family income	<2000	273 (66.4%)
	≥2000	138 (33.6%)
Family size	≤4	167 (40.6%)
	>4	244 (59.4%)
Age of the child in month	36–47	252 (61.3%)
	48–59	159 (38.7%)
Sex of the child	Male	196(47.7%)
	Female	215(52.3%)
Number of under 5 children within home	≤2	401(97.6%)
	>2	10 (2.4%)

### Health and nutrition-related characteristics of the participants

**Figure 1:**
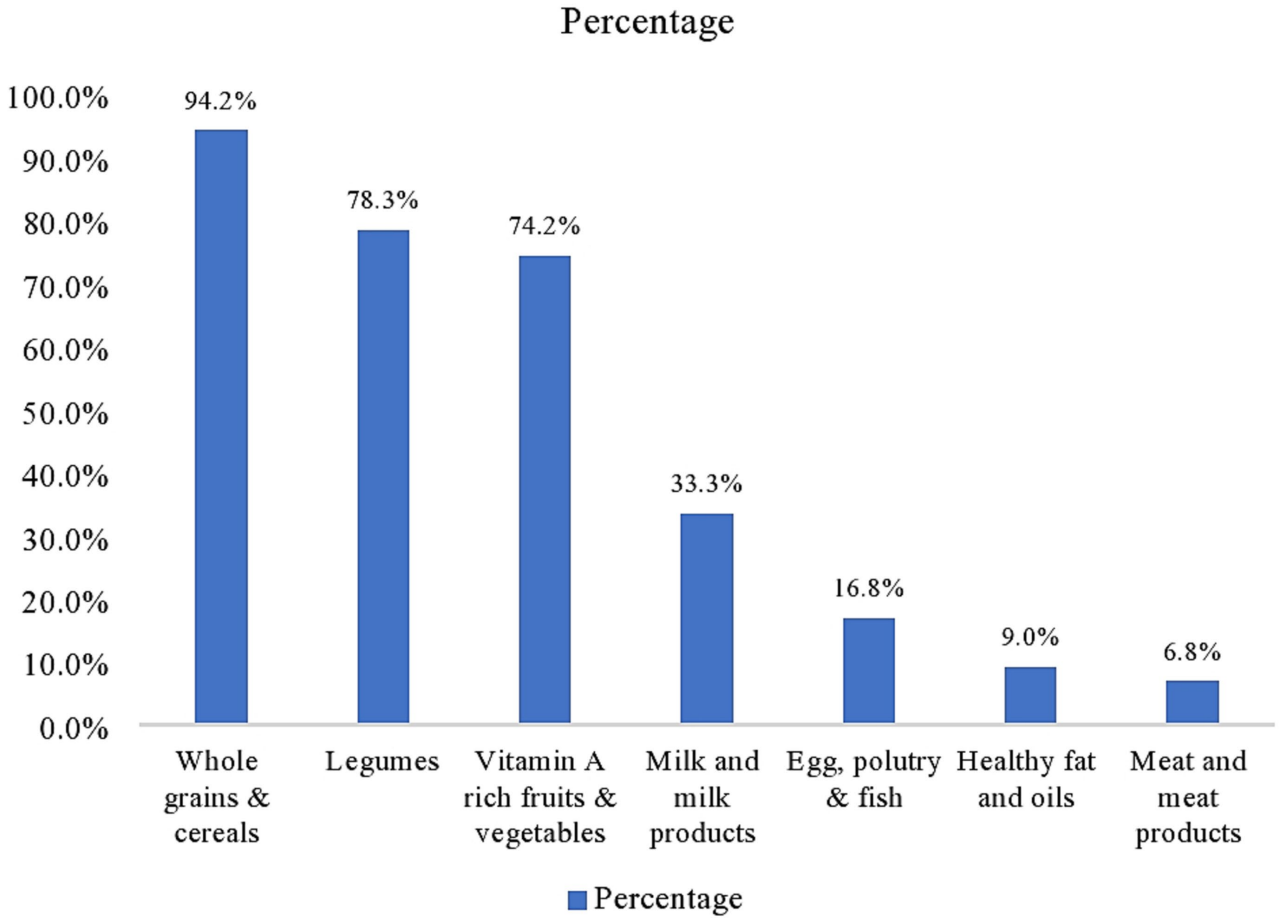
Proportion of children 3–5 years old who consumed the seven food groups in last 24 h preceding the survey, Cheha district, Gurage Zone, southern Ethiopia, 2023.

Most of the mothers (361; 87.8%) had attended antenatal care (ANC) visits for their children. About 350 (85.2%) mothers reported that their children had received vitamin A capsule supplementation, and out of the 350 children, only 188 (53.7%) took the vitamin A capsule supplementation in the last 6 months. Regarding the nutritional status, 18.5%, 16.1%, and 13.6% of the children were stunted, wasted, and underweight, respectively. Most children (84.2%) had inadequate dietary diversity scores. Moreover, 94.2 and 78.3% of the children ate wholegrain and legumes, respectively, in the last 24 h preceding the survey. About three-fourths (304) of the respondents cultivate vitamin A rich vegetables in their garden (Table 2; Figure 1).

**Table 2:** Health and nutrition related characteristics of study participants in Cheha District, southern Ethiopia, 2023.

Characteristics	Frequency (%)
ANC follow up	
Yes	361 (87.8%)
No	50 (12.2%)
Full immunization	
Yes	355 (86.4%)
No	56 (13.6%)
Took vitamin A capsule	
Yes	350 (85.2%)
No	61 (14.8%)
Time of receiving last dose of vitamin A capsule	
≤6 months	188 (53.7%)
>6 months	162 (46.3%)
Child ill 1 week preceding the survey	
Yes	120 (29.2%)
No	291 (70.8%)
Stunting	
Yes	76 (18.5%)
No	335 (81.5%)
Underweight	
Yes	66 (16.1%)
No	345 (83.9%)
Wasting	
Yes	56 (13.6%)
No	355 (86.4%)
Dietary diversity	
Adequate	65 (15.8%)
Inadequate	346 (84.2%)
Cultivate vitamin A rich vegetables	
Yes	306 (74.5%)
No	105 (25.5%)

### Prevalence of clinical VAD

**Figure 2:**
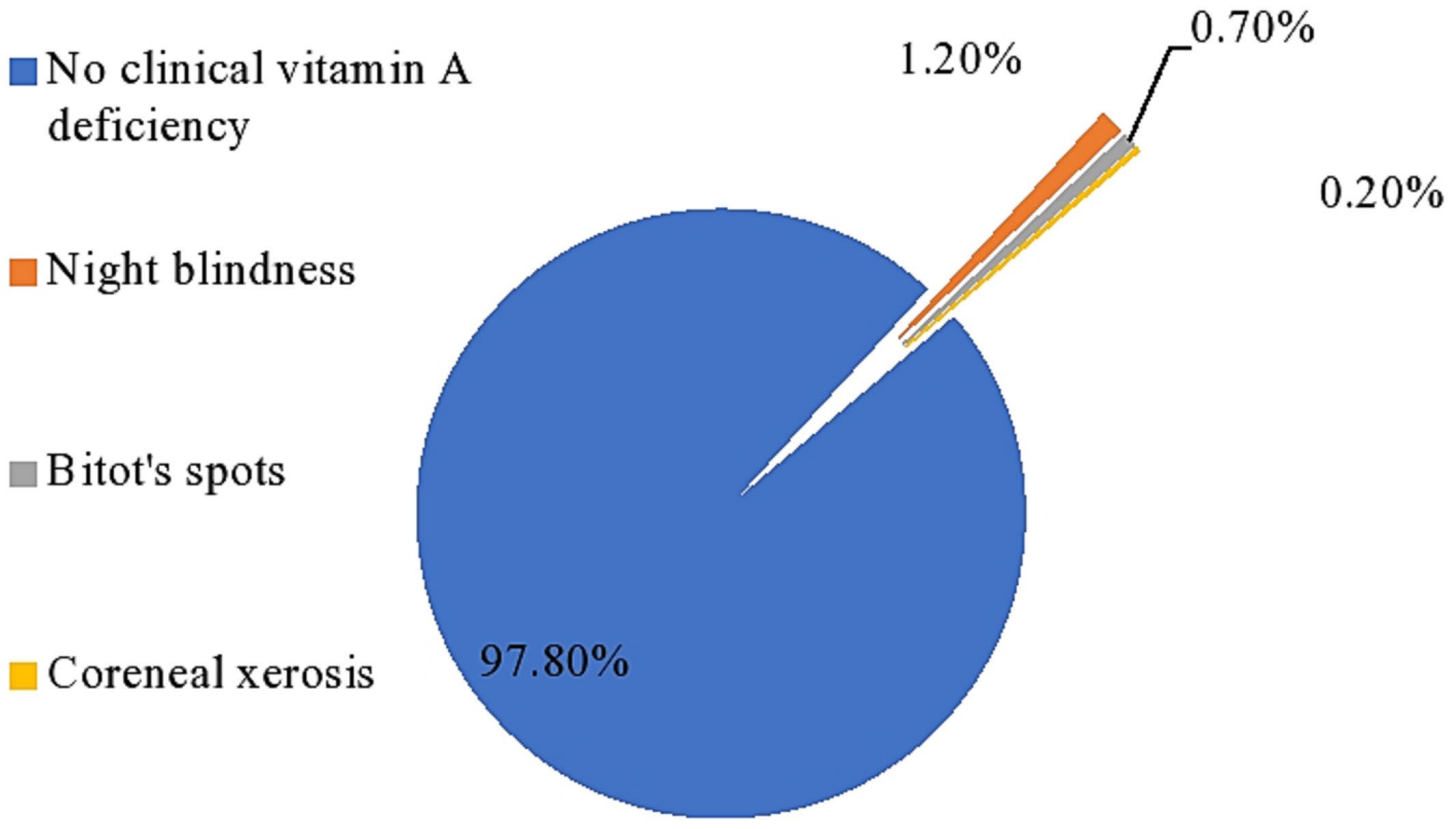
Prevalence of clinical vitamin-A deficiency among PSC in Cheha district, southern Ethiopia, 2023.

The overall prevalence of clinical vitamin A deficiency in the study area was 2.2% (95% CI: 1.7–2.7%). When disaggregated by the clinical form, night blindness affects 1.2%, bitot’s spots affects 0.7%, and corneal xerosis affects 0.2%, indicating a major public health problem compared to the WHO cutoff point (Figure 2).

### Factors associated with clinical VAD

In the bivariable analysis, the age of the child, vitamin A capsule supplementation, ANC visit, and educational status of the mothers were significantly associated with clinical VAD. The variables that had a *p*-value of 0.25 in the bivariable analysis and relevant determinants of VAD from previous studies were considered for the multivariable logistic regression to adjust for potential confounders and identify independent factors affecting the odds of VAD. The model’s fitness was checked by Hosmer and Lemeshow’s test, and it was non-significant with a *p*-value of 0.95, which indicates that the model is fit. Hence, in the crude analysis, without considering potential confounders, the age of the child, the mother’s education, not having ANC visits, and getting VAS were significant determinants of VAD among PSC.

This study revealed the odds of developing clinical VAD are higher among PSC aged 48–59 as compared to 36–47-month-old children (AOR = 17.3; 1.89–158.7). Moreover, the risk of having a VAD child was higher in households with more than two under-five-year-old children, indicating the potential role of short birth spaces and a larger family size for VAD. The risk of clinical VAD was shown to be concentrated among older children without VAS. Children from mothers who had ANC visits were significantly associated with reduced odds of clinical VAD (AOR = 0.02; 0.001–0.32) as compared to their counterparts. The odds of the occurrence of clinical VAD were 91% lower among children who received VAS (AOR = 0.09; 0.01–0.70) without reporting VAS. On the other hand, the odds of developing clinical VAD were 5.5 times higher among children without growing vegetables in the backyard (AOR = 5.31; 0.34–83.2), and 9 times higher among children with lower estimated monthly income (AOR = 9.39; 0.64–138.7). The occurrence of clinically evident VAD is closely determined by income, the availability of vitamin A-rich foods, and not getting routine VAS. More importantly, having inadequate dietary diversity is associated with higher odds of developing VAD (AOR = 5.50; 0.37–82.6). The same association was also explored, where stunted children (AOR = 2.49; 0.41–15.0) had 2.5-fold increased odds of developing VAD as compared to non-stunted children (Table 3).

**Table 3:** Logistic regression analysis for determinants of clinical VAD among children in southwest Ethiopia 2023.

Variables		Clinical VAD		COR (95% CI)	AOR (95% CI)	*p*-value
		Yes	No			
Maternal age	≤35	7	337	1	1	
	>35	2	65	1.48 (0.30–7.29)	3.63 (0.38–34.3)	0.261
Child sex	Male	5	191	1.38 (0.36–5.22)		
	Female	4	211	1		
Age of the child	36–47	2	250	1	1	
	48–59	7	152	5.76 (1.18–28.1)	17.3 (1.89–158.7)	0.012*
Educational status of the mother	Illiterate	7	159	5.35 (1.10–26.08)	9.19 (0.96–88.4)	0.055
	literate	2	243	1	1	
Family size	<=4	2	165	1		
	>4	7	237	2.44 (0.50–11.9)		
Under-five children	≤2	8	393	1	1	
	>2	1	9	5.46 (0.62–48.4)	16.2 (0.81–323.1)	0.069
Illness in the past week	Yes	5	115	3.12 (0.82–11.8)		
	No	4	287	1		
Underweight	Yes	2	64	1.51 (0.31–7.43)		
	No	7	338	1		
Wasting	Yes	1	55	1.27 (0.16–10.3)		
	No	8	347	1		
Income	<2000	8	265	4.14 (0.51–33.4)	9.39 (0.64–138.7)	0.103
	≥2000	1	137	1		
Stunting	Yes	4	72	3.67 (0.96–13.9)	2.49 (0.41–15.0)	0.320
	No	5	330	1	1	
Dietary diversity	Adequate	1	64	1	1	
	Inadequate	8	338	1.52 (0.19–12.3)	5.50 (0.37–82.6)	0.217
Growing vegetables in the backyard	Yes	4	302	1	1	
	No	5	100	3.78 (0.99–14.3)	5.31 (0.34–83.2)	0.235
ANC visit	Yes	3	358	0.06 (0.02–0.25)**	0.02 (0.001–0.32)	0.005**
	No	6	44	1	1	0.005*
Vitamin A supplementation	Yes	3	347	0.08 (0.02–0.33)**	0.09 (0.01–0.70)	0.021*
	No	6	55	1	1	

The symbol asterisks refer to statistically significant determinants of VAD at *p*-value below 0.05 (*) and 0.01(**).

## Discussion

This study tried to depict the prevalence of clinical VAD and associated factors among PSC aged 3 to 5 years. The overall prevalence of clinical VAD in the study area was 2.2% (1.7–2.7%), which represents a major public health problem according to the WHO cut-off point for public health significance for PSC, which is ≥1.56% (12). Concerning the occurrence of night blindness, bitot’s spots are also higher as compared to the WHO recommendations of 1%, 0.5%, and 0.01%, respectively. This could be attributed to low intake of vitamin A-rich foods and low effective coverage of routine VAS (7). Other studies in the south Gondar zone and north Ethiopia showed that 2 and 2.6% of children had VAD, respectively (27, 34). It was also similar to the studies done in India (35) and Yemen (36).

On the other hand, a higher prevalence of VAD is reported in northwest Ethiopia (8.6%) (28). These variations could be associated with basic variations in demographic factors, socioeconomic classes, VAS coverage (37, 38), and access to and consumption of vitamin A-rich fruit and vegetables. Hence, this emphasizes the spatial variation in the risk of VAD among different geographic locations, driven by local factors (21). Even though routine VAS is in place in Ethiopia, VAD is still a major public health problem that needs to be considered due to the variation in VAS coverage. Enhanced consumption of animal-source foods with a better bioavailability and conversion rate to retinol is needed to tackle VAD. Hence, diversifying our diet is a crucial intervention (39). In addition, intervention measures to increase economic access to vitamin A-rich food sources like OFSP, animal-source foods, and vitamin A-rich fruits and vegetables could improve vitamin A nutrition and its health outcomes. It should be noted that the retinol conversion efficiency of plant-source foods is very limited (9).

The study showed that the odds of developing clinically evident VAD among children aged 36–47 months were relatively lower compared to older children. The finding is consistent with a study from Ethiopia (28), Sudan (40), and India (36). In fact, children aged 36–47 months might be susceptible to VAD since it is a time for the cessation of breast feeding and diversified dietary consumption could be very limited. However, they tend to be asymptomatic in the early stages, as it takes time to develop clinically evident VAD. The risk is expected to be higher among this age group, yet they have hidden VAD. This could be further explained by the higher coverage of VAS for younger children compared to older children. However, a study from northwest Ethiopia (41) showed the reverse. Overall, PSC are very susceptible to VAD, yet the risk might vary by age group, driven by the occurrence of major childhood illnesses like diarrhea, pneumonia, and measles.

Furthermore, the educational status of the mother, rural residence, and family size are important predictors of clinical VAD (34, 35, 42). This might be due to the fact that the number of interviewed mothers who were literate was relatively higher in this study (40.4%) compared to the other study and about two-thirds of the respondents had a family size of >4, which makes most of the respondents fall into that category. In addition, those variables might be the predictors of subclinical levels of VAD rather than clinical VAD, which is mainly based on serum retinol levels. Hence, the findings of this study would guide targeted interventions to tackle VAD through integrated health and nutrition interventions. For instance, growing vegetables in the backyard could help increase access to vitamin A-rich foods.

The current study found that there is no statistically significant gender-based difference in developing clinical VAD among PSC (COR = 1.38; 0.36–5.22). This was supported by the findings of the study done in Ethiopia (27). However, it was contradicted by reports from other studies in Ethiopia, which showed that males are more susceptible to clinical VAD than females (28, 34). This has been indicated by Israel (43) and India (35). Basically, there might be some baseline differences in immunity, susceptibility to infections, and gender-based feed preference (44). The difference might be related to the slightly higher nutritional requirements of male children, so, they might be highly susceptible to micronutrient deficiency and major childhood illnesses as they tend to be exposed to an external environment.

Additionally, not having ANC follow for the mother was an important risk factor for prevailing VAD among older children. Although routine VAS is not practiced for pregnant women, having ANC follow-up during maternal pregnancy is a baseline for providing nutritional care and counseling and increasing consumption of vitamin A-rich foods (45). This could help ensure adequate postnatal maternal retinol storage and breast milk for the child. This would also encourage the likelihood of receiving other maternal and child health services, and postnatal VAS would help reduce the risk of VAD (46). The finding was consistent with most of the studies conducted in Ethiopia and outside Ethiopia (20, 22, 28). Hence, strengthening the effective utilization of ANC is crucial for maternal and child survival.

Given the effective role of routine VAS, this study also supports the fact that the risk of VAD was 82% lower among those who had routine VAS. This was supported by studies done in Ethiopia and India (2, 22, 28), emphasizing the need for enhanced VAS coverage and targeting vulnerable segments of the population. This might be due to the fact that vitamin A supplementation has been proven effective in reducing the impacts of both clinical and subclinical VAD, particularly among children six months to five years of age (38).

Beyond these, not having growing vegetables in the backyard, children with an inadequately diversified diet, and stunting increase susceptibility to VAD. The former two are closely related to each other, and availability could improve dietary diversity and ultimately access to micronutrient intake (4, 46). On the other hand, being stunted is associated with long-term micronutrient deficiency starting in the uterus, which could increase the risk of VAD. On the other hand, VAD could lead to growth failure and stunting. This emphasizes the need for counseling for dietary diversification and enhancing the production of locally produced fruits and vegetables, which could reduce VAD among children (47, 48). A study from Uganda showed that VAD increases the odds of stunting by 43% (48). However, access to a diversified diet and stunting had a strong discrepancy among different socioeconomic classes (47).

This study has generated new evidence in the study area on the risk factors of VAD in southwest Ethiopia, which has not been investigated before in the study area. However, these findings should be sought in light of some limitations. The clinical assessment to depict VAD only shows the tip of the iceberg, omitting subclinical VAD. These should usually be captured using serum retinol, and the prevalence would be very high. Recall bias and social desirability bias in assessing dietary intakes over the past 24 h could not be excluded. It might have been a risk of recall bias as dietary assessments were made through a 24-h recall, which has a risk of socially desirable bias from the respondent’s side. Due to the rare nature of the outcome, the current sample size could not be adequate hence limiting its representativeness and statistical power. This could impact the overall prevalence estimate of clinical VAD.

## Conclusions and recommendations

The overall prevalence of clinical VAD among PSC in this study area is a major public health concern that needs enhanced VAS, nutrition education and counseling, and other targeted nutrition-sensitive interventions. The ANC visit of the mother, vitamin A supplementation status, and age of the child were factors that determined clinically overt VAD. Therefore, awareness should be given to the community by primary health care workers, including health extension workers, about dietary sources and prevention methods of VAD. The implementation of postnatal VAS for better childhood vitamin A status should be emphasized. This behavioral intervention should be enhanced during the ANC visits. In addition, the zonal health department and woreda health office should strictly check the implementation status of VAS in the district. Moreover, further investigation of subclinical vitamin A deficiency using reliable biomarkers and dietary intake data with a larger sample size is strongly recommended to inform better decisions.

## Data availability statement

The original contributions presented in the study are included in the article/supplementary material, further inquiries can be directed to the corresponding author.

## Ethics statement

The studies involving humans were approved by Wolkite University Institutional Review Board. The studies were conducted in accordance with the local legislation and institutional requirements. Written informed consent for participation in this study was provided by the participants’ legal guardians/next of kin.

## Author contributions

AN: Conceptualization, Data curation, Formal analysis, Funding acquisition, Investigation, Methodology, Project administration, Resources, Software, Supervision, Validation, Visualization, Writing – original draft, Writing – review & editing. TM: Supervision, Visualization, Writing – review & editing. AA: Conceptualization, Data curation, Formal analysis, Methodology, Project administration, Software, Supervision, Validation, Visualization, Writing – original draft, Writing – review & editing.
